# Structural characterization and duplication modes of pseudogenes in plants

**DOI:** 10.1038/s41598-021-84778-6

**Published:** 2021-03-05

**Authors:** Flavia Mascagni, Gabriele Usai, Andrea Cavallini, Andrea Porceddu

**Affiliations:** 1 Department of Agricultural, Food, and Environment, University of Pisa, Via del Borghetto 80, 56124 Pisa, Italy; 2 Dipartimento di Agraria, Università degli studi di Sassari, Via Enrico de Nicola 1, 07100 Sassari, Italy

**Keywords:** Comparative genomics, Plant evolution

## Abstract

We identified and characterized the pseudogene complements of five plant species: four dicots (*Arabidopsis thaliana*, *Vitis vinifera*, *Populus trichocarpa* and *Phaseolus vulgaris*) and one monocot (*Oryza sativa*). Retroposition was considered of modest importance for pseudogene formation in all investigated species except *V. vinifera*, which showed an unusually high number of retro-pseudogenes in non coding genic regions. By using a pipeline for the classification of sequence duplicates in plant genomes, we compared the relative importance of whole genome, tandem, proximal, transposed and dispersed duplication modes in the pseudo and functional gene complements. Pseudogenes showed higher tendencies than functional genes to genomic dispersion. Dispersed pseudogenes were prevalently fragmented and showed high sequence divergence at flanking regions. On the contrary, those deriving from whole genome duplication were proportionally less than expected based on observations on functional loci and showed higher levels of flanking sequence conservation than dispersed pseudogenes. Pseudogenes deriving from tandem and proximal duplications were in excess compared to functional loci, probably reflecting the high evolutionary rate associated with these duplication modes in plant genomes. These data are compatible with high rates of sequence turnover at neutral sites and double strand break repairs mediated duplication mechanisms.

## Introduction

Pseudogenes are genic derived sequences that have lost the capability to encode a functional protein^1–3^. Based on the intron-exon structure they are classified into two major types: non processed (or duplicated) and processed (or retroposed). Non processed pseudogenes arise from genome or chromosomal duplications and usually retain the exon-intron structure of ancestral genes^4^. Processed pseudogenes derive by genomic integration of reverse transcribed messenger RNA, are typically devoid of introns, presenting flanking direct repeats and a poly-adenine tail at their 3’ end^2,5^. The functional paralogous showing the top sequence and structural homology to a pseudogene is referred to as “parental” locus or the youngest pseudogene prototype^3,6^. Although defunct copies of functional genes, pseudogenes have recently stimulated a wide range of scientific interests ranging from structural and evolutionary genomics^7^ to post-transcriptional gene expression regulation studies^3,8,9^. Comparative analyses of the pseudogenes have proved informative for both genome and gene evolutionary studies. For example, the balance between pseudogenes types has indicated different modes of gene amplification between plants and mammalians. The high prevalence of processed over non processed pseudogenes in mammalians genomes has been proved in sharp contrast with the low ratios of processed versus non processed types in plants^10,11^. Non collinear sequence duplication would occur mainly by retro-positions in mammalians^3,11^ while double strand break repair connected mechanisms would be prevalent in plants^10,12^. Pseudogenes have been used also to infer past genomic organization and gene family evolution. The high abundance of pseudogenes belonging to specific gene families has been used as an indication of low selective pressure on genic functions that are no longer important for the fitness of one species. To cite an example, *H. sapiens* presents a high number of olfactory receptor pseudogenes indicating that, during evolution, the functions exerted by these genes have lost importance in the hominids^13^. However, in other cases, pseudogene copy number correlates with the expression level of the functional parental gene^14^. Intraspecific analysis of pseudogenes have revealed how they contribute to genome plasticity by favoring sequence diversity. Indeed, as a consequence of low selective pressure, pseudogenes show a high propensity to accumulate mutations which can be transferred to functional genes by gene conversion mediated events^1^. Zhu et al. ^15^ have demonstrated that conversions involving pseudogenes could accelerate gene family evolution and divergence relative to their orthologs. Gene conversion has been cited by Prade et al. ^10^ to explain the positive association between physical distance and sequence diversity of pseudogene parental-pairs. The interest for pseudogene has recently gained new emphasis following the demonstrations that they may play a role in regulating the expression of their functional cognate^16,17^. A recent study on seven plant genomes showed that a surprisingly large fraction of non transposable element (TE) and regulatory non coding RNAs (microRNA and long non coding RNAs) originate from transcription of pseudogene proximal upstream regions^7^. Based on these observations it was proposed that the recruitment of pseudogene-derived regulatory sequences may lead to the origin of novel regulatory modules^7^. Comparative analyses of pseudogene complements have been demonstrated very informative in illustrating how remodelling processes mark each organism genome evolution. For example, the comparison of pseudogene complements across *H. sapiens*, *C. elegans* and *D. melanogaster* phyla highlighted lineage specific features capable of reflecting genome histories more than the conservation of essential biological functions^18^. Specifically, the human pseudogene complements illustrated a massive burst of retroposition activity at the dawn of the primates while the fly and worm reflected a history of deactivated duplications. Most of our knowledge on plant genome evolution has been gained through comparative analysis of structural features of functional gene complements, but yet very little emphasis has been dedicated to the analysis of pseudogene complements. Exploiting the genomic resources publicly available, here we present a comparison of structural features of the pseudogene complements of five species: four dicots (*A. thaliana*, *P. vulgaris*, *P. trichocarpa*, *V. vinifera*) and one monocot (*O. sativa*). The four dicots were chosen to represent different vegetative habitus and lifespan of the two main groups of rosids: Vitales and eurosids. *V. vinifera* is representative of the order Vitales, and shows a woody habitus and a perennial lifespan. The other species belong to the main orders of eurosids: the *Fabidae* with the annual and herbaceous species *P. vulgaris*, the *Malvidae* with the perennial and woody *P. trichocarpa* and the annual and herbaceous *A. thaliana*. Our analysis confirmed that processed are less abundant than non processed pseudogenes in all investigated species but highlighted also a significant variation for the relative abundance and genomic distribution of pseudogene types which reflected lineage specific differences. Furthermore, although the majority of plant pseudogenes seems to derive from duplicative transpositions, the analysis of pseudogene-parental locus genomic positions reflected evolutionary differences for the prevalent modes of pseudogene origin among the investigated species.

## Results

**Figure 1:**
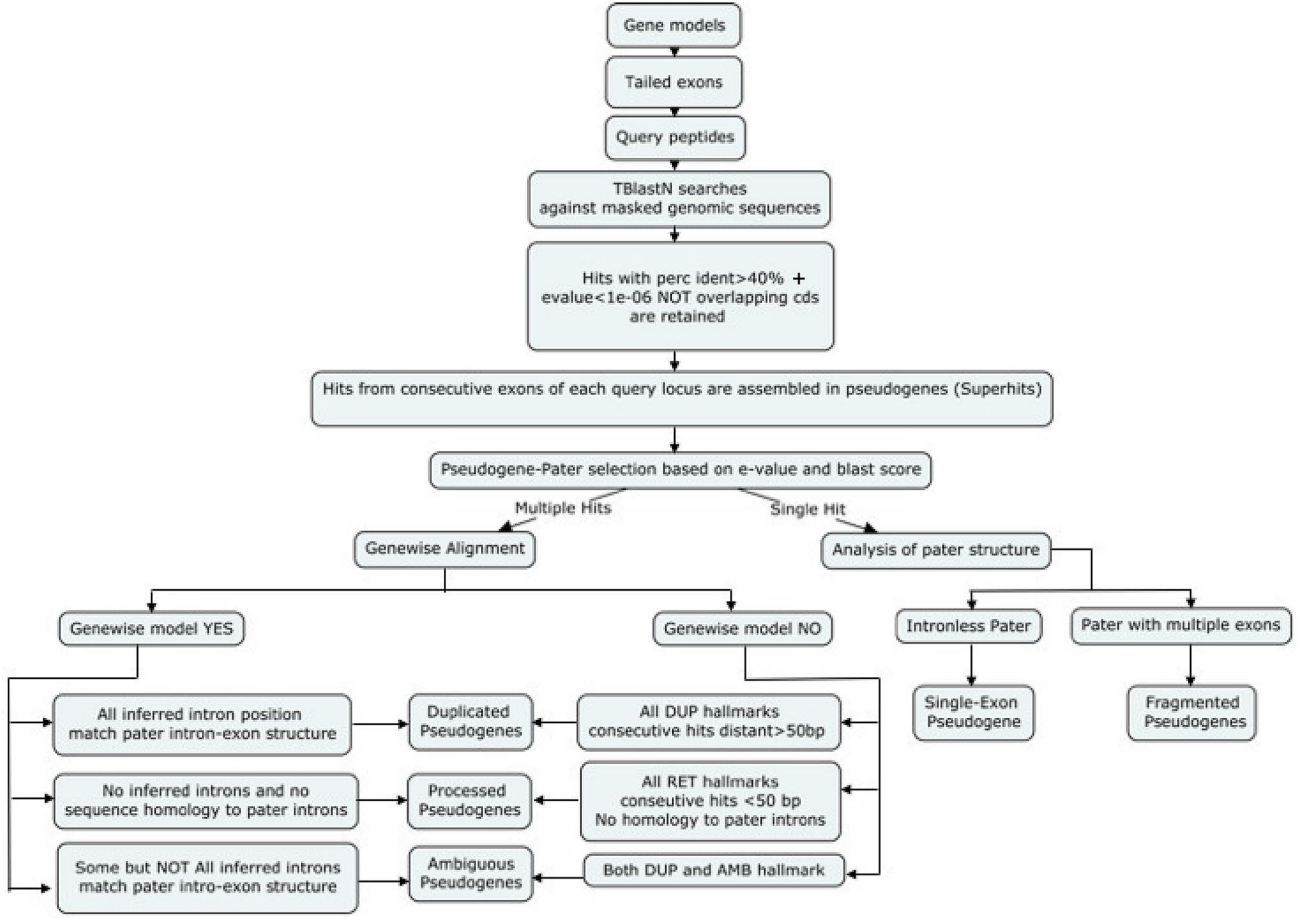
Procedures followed to identify and classify the pseudogenes based on their inferred intron-exon structures. *DUP* duplication, *RET* retroposition, *AMB* ambiguous.

**Figure 2:**
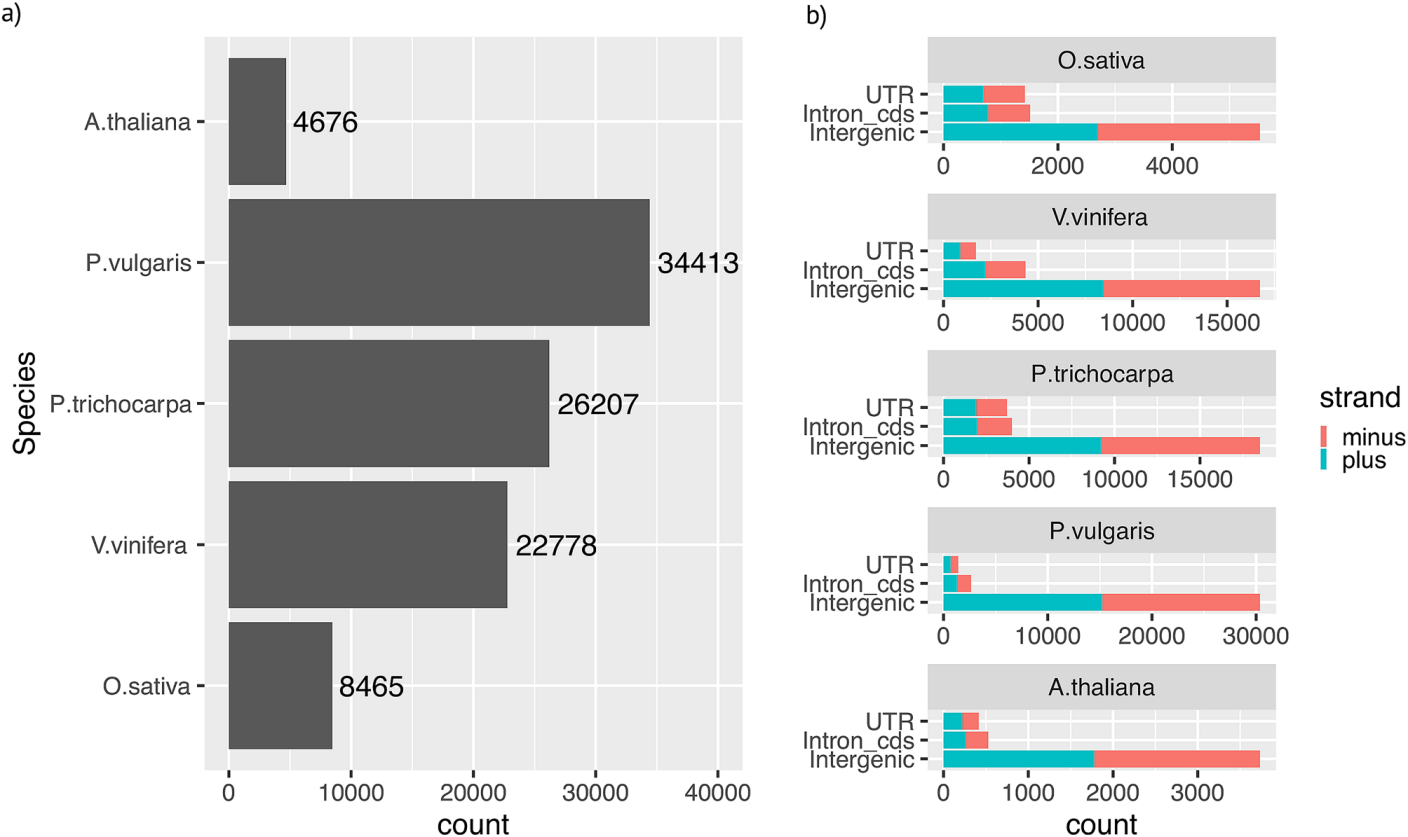
Numbers and distributions of pseudogenes in the five investigated species. (**a**) Total numbers of pseudogenes identified in each species. (**b**) Pseudogene distributions based on their positions in either DNA strands. *UTR* untrnslated 5’, 3’ exons of genes and introns therein, *Intron*_*cds* intron of the coding sequences, *Intergenic* pseudogenes that map between two consecutive genes.

Pseudogenes were identified based on sequence homology to functional loci in *Arabidopsis thaliana*, *Populus trichocarpa*, *Phaseolus vulgaris*, *Vitis vinifera* and *Oryza sativa* genomes. The pipeline used for pseudogene identification and classification is represented in Fig. 1. Translated coding exons of each functional locus were used to query the hard-masked genomic sequences by tBlastN^19^. Hits not overlapping coding sequence coordinates and matching consecutive exons of query loci were merged to identify putative pseudogenes. When a genomic region matched overlapping pseudogene models, only the model with the highest homology was chosen for further analysis (Fig. 1). This criterion derives from the hypothesis that the functional homologous showing the highest sequence identity to the pseudogene sequence is the best extant version of the parental locus^6^. Pseudogene models were predicted by aligning the query protein (encoded by the inferred parental locus) to the pseudogene genomic sequence. When introns were predicted at all the positions expected based on the model of the query locus, the pseudogenes were classified as non processed. If no introns were predicted, though expected based on the parental gene model, the pseudogenes were classified as processed. Pseudogenes with models showing features of both duplicated and processed were classified as ambiguous. If the alignments did not cover intron positions the pseudogenes were classified as fragmented or single exon if identified by a multi-exon or single-exon parental, respectively. In total, we identified 74,874 pseudogenes within intergenes (41,684 with disablements), 13,007 within introns of the coding sequences (5,289 with disablements) and 8,658 within untranslated transcribed regions (3705 with disablements) (Fig. 2a; Supplemental Table S2). Pseudogenes showed similar relative abundance within these regions and for their orientation in DNA strands among the investigated species (see Fig. 2b).

### Processed pseudogenes are the minority in plant genomes

**Figure 3:**
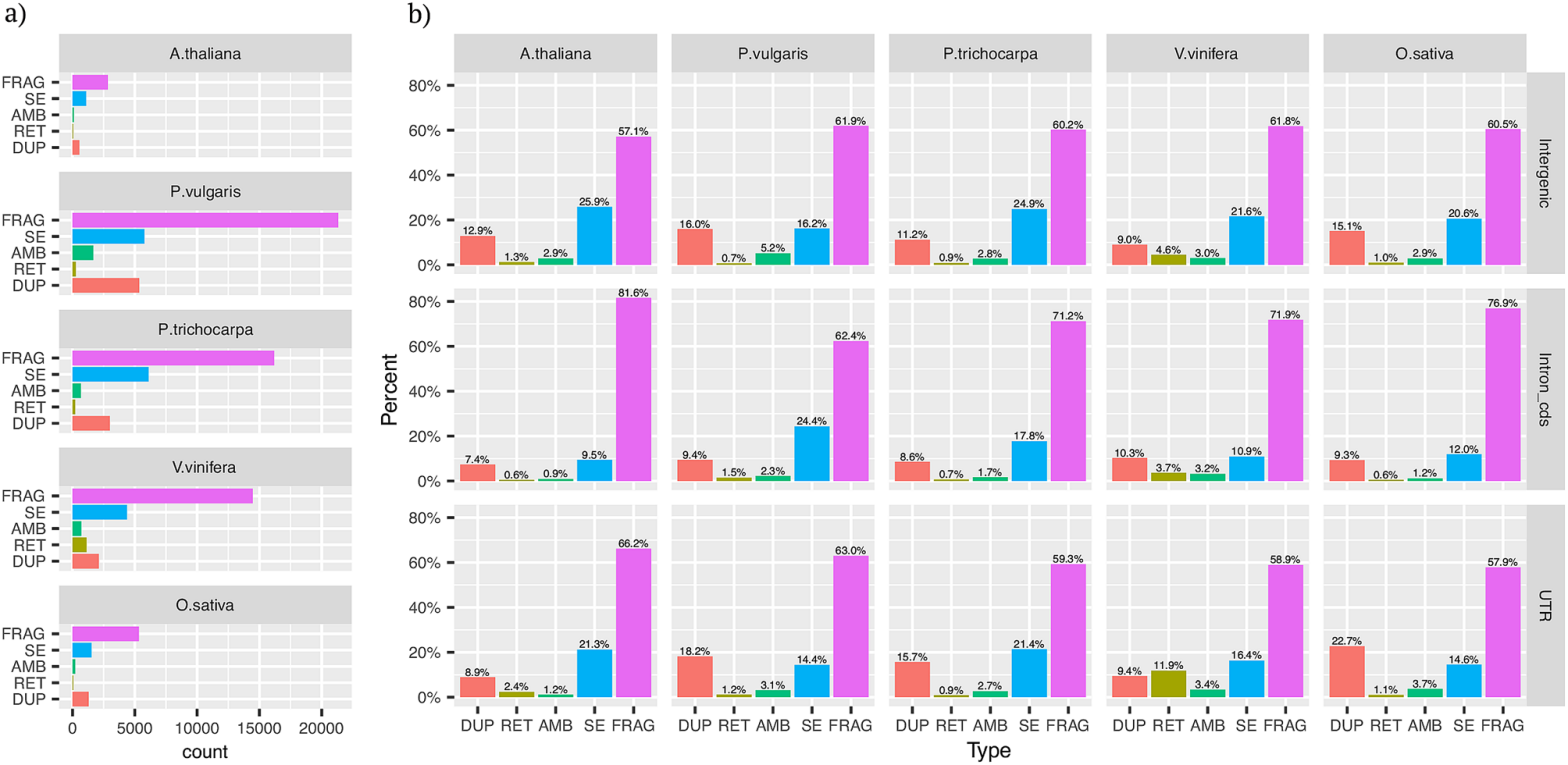
Distributions of pseudogenes according to presence/absence of introns (**a**) Total number of peudogenes types for the five investigated species. (**b**) Distribution of pseudogenes (as relative frequencies) among the different genomic regions. *FRAG* fragmented, *SE* single-exon pseudogenes, *AMB* ambiguous pseudogenes, *RET* processed psedupogenes.

Fragmented and single-exons were the most abundant pseudogenes in all analyzed species (Fig. 3 and Supplemental Tables S2 and S3). The ratio between non processed and processed pseudogene types marked relevant variations among species. Non processed were tenfold more abundant than processed pseudogenes in *P. trichocarpa*, *P. vulgaris*, *A. thaliana* and *O. sativa* and about twofold more abundant in *V. vinifera* (Supplemental Table S2 and S3a and Fig. 3). These ratios were not affected by differences in genic architectures of parental loci. Indeed, both the relative number of parental loci with multiexon models (Supplemental Figure S2) and the proportion of parental exons kept in pseudogenes models showed little variation between species (Supplemental Figure S3). The distributions of pseudogene types within genic regions inferred from gene predictions (Untranslated regions, introns of the cds and intergenes) reflected the overall pattern of pseudogene types (Fig. 3a, b) except for *V. vinifera* UTRs which showed more processed than non processed pseudogenes. The investigated species were also distinguished for the distribution of the number of pseudogenes identified by each parental locus (see Supplemental Figure S3). *A. thaliana*, *P. trichocarpa* and *O. sativa* showed 48%, 27% and 35% of parental loci each identifying a single pseudogene and, 1.13%, 2.3% and 1.7% of parental loci each identifying more than five pseudogenes. 8.7% and 17.3% of parental loci matched a single pseudogene in *P. vulgaris* and *V. vinifera* and 2.2% and 3.5% more than five pseudogenes. Based solely on intron-exon structure it is very difficult to infer if an intron-less pseudogene derived from (primary) retroposition of a multi-exon parental locus or genomic duplication of a retroposed parental paralog (secondary retroposition). These cases are particularly uncertain when the duplications are separated by a short time-lapse. It is therefore possible that some SE pseudogenes are misclassified as retro-pseudogenes. To evaluate the possible impact of these cases on our conclusions we reanalyzed parental locus assignment for all pseudogenic regions which matched both multi-exon and single exon paralogous (see Table S3b). The prevalence of duplicated over transposed pseudogenes was confirmed for all investigated species even when all dubious parental locus assignments were resolved in favour of the multi-exon candidate.

### The pseudo and functional gene complements show different duplication mode preferentiality

**Figure 4:**
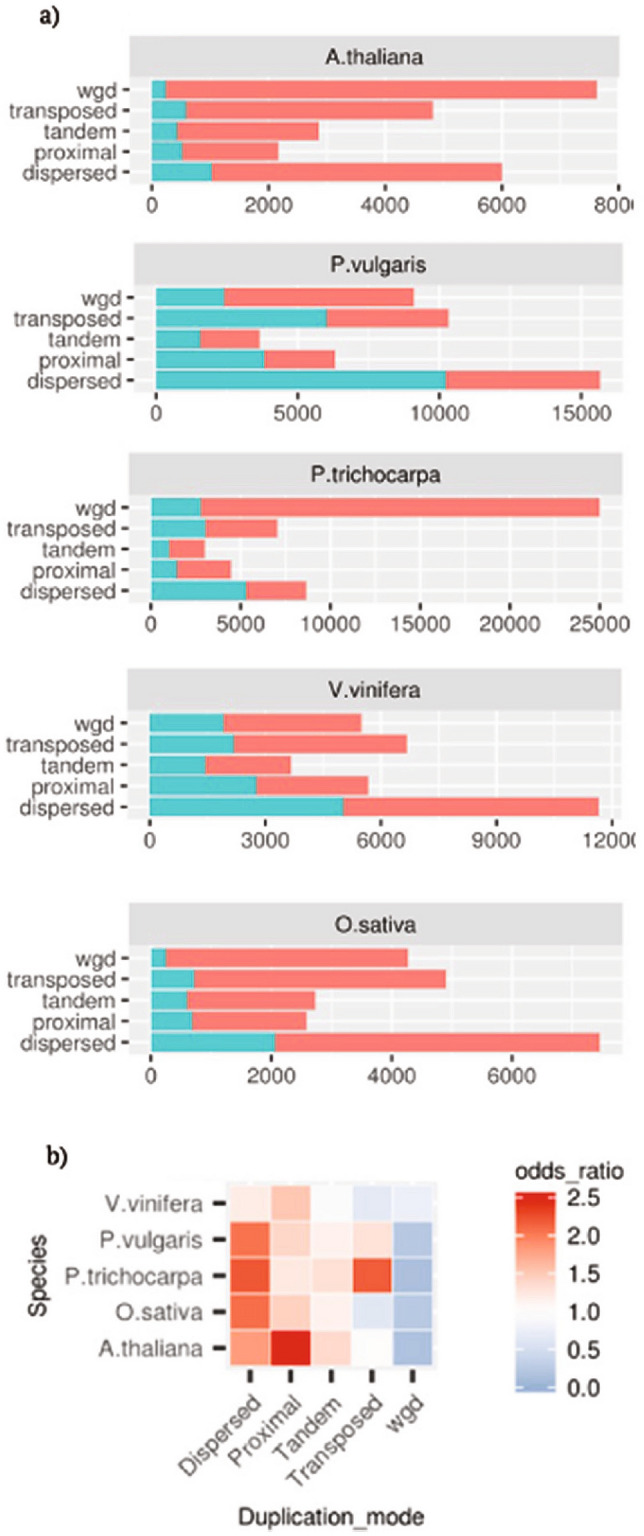
Pseudogenes (blue) and functional loci (red) classifications across the five investigated species based on analysis of the maps of collinearity. (**b**) Odds ratios for the proportions of pseudogenes versus functional loci based on the defined mode of duplication. Significance of odds ratio>1.0 or<1.0 tested by Fisher’s exact tests; white squares, *P*>0.05.

The modes of sequence duplications were inferred by interrogating genomic intra and inter species maps of collinearity^20^. To avoid possible conflicts deriving from overlapping coordinates (for example between functional loci and pseudogene eventually mapping within introns or UTRs) only pseudogenes mapping within inter-genes were considered for these analyses. In case a given locus was attributed to more than a duplication mode its final classification was chosen based on the following priority wgd> tandem> proximal> transposed> dispersed. Pseudogenes showed a proportionally higher tendency than functional loci (Fisher’s exact test; *P*< 0.05) to be classified as dispersed or proximal (Fig. 4a, b). On the contrary, there were proportionally less wgd pseudogenes than wgd functional loci. This observation holds also for *P. trichocarpa* which due to the recent whole genome duplication of *Salicaceae* showed an high proportion of wgd duplications. Tandem duplications were proportionally more frequent in the pseudo than in the functional gene complement in all analyzed species except *V. vinifera*. The transposed duplication mode marked the highest differences among species. If compared with functional loci, pseudogenes were proportionally enriched for transposed duplicates in *P. trichocarpa* and *P. vulgaris* and vice-versa in *V. vinifera* and *O. sativa*.

### Relations between pseudogene structure and origin

**Figure 5:**
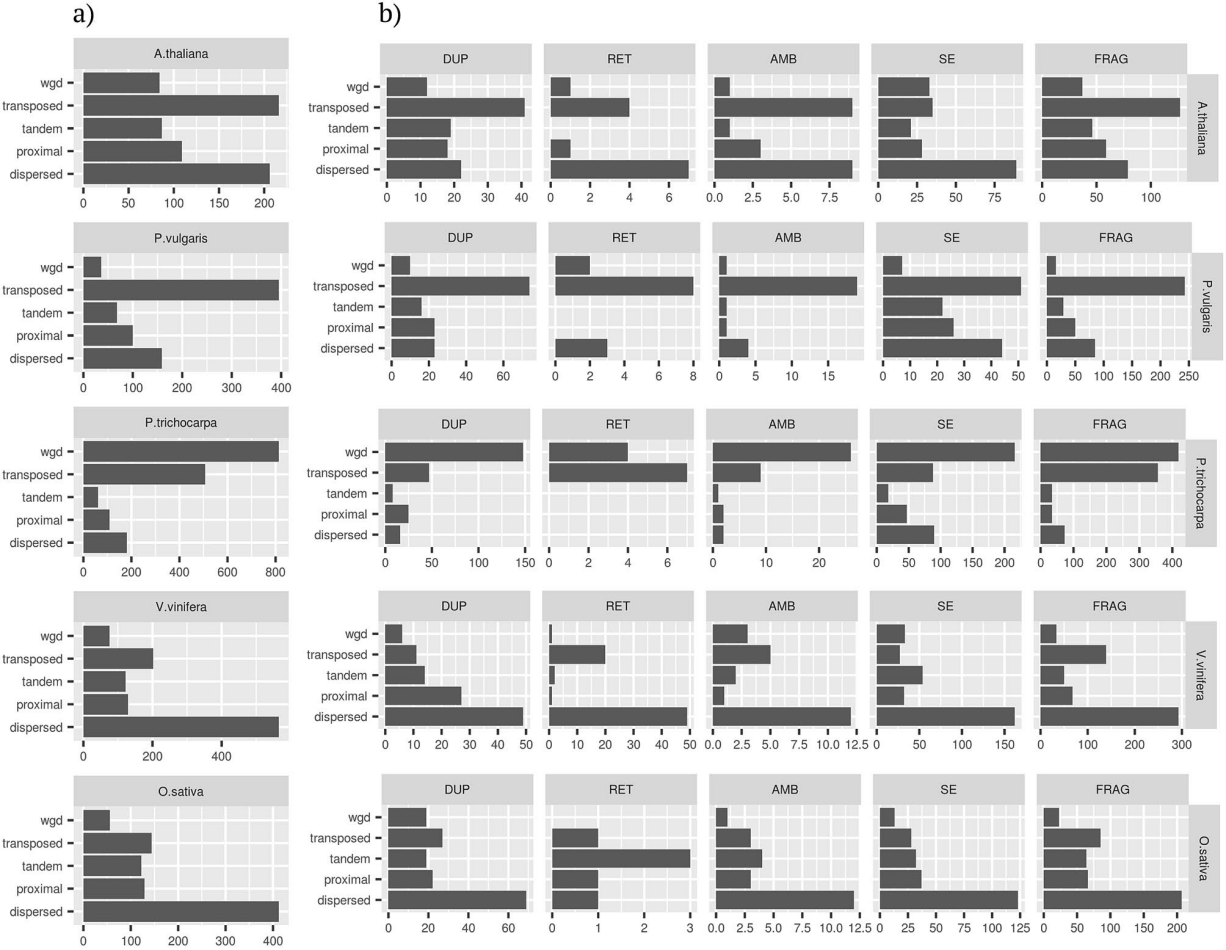
Pseudogene classification based on both intron exon structure and duplication modes in the five analyzed species. (**a**) Classification based on the duplication mode only. (**b**) Classification based on both duplication mode and intron exon structure.

**Figure 6:**
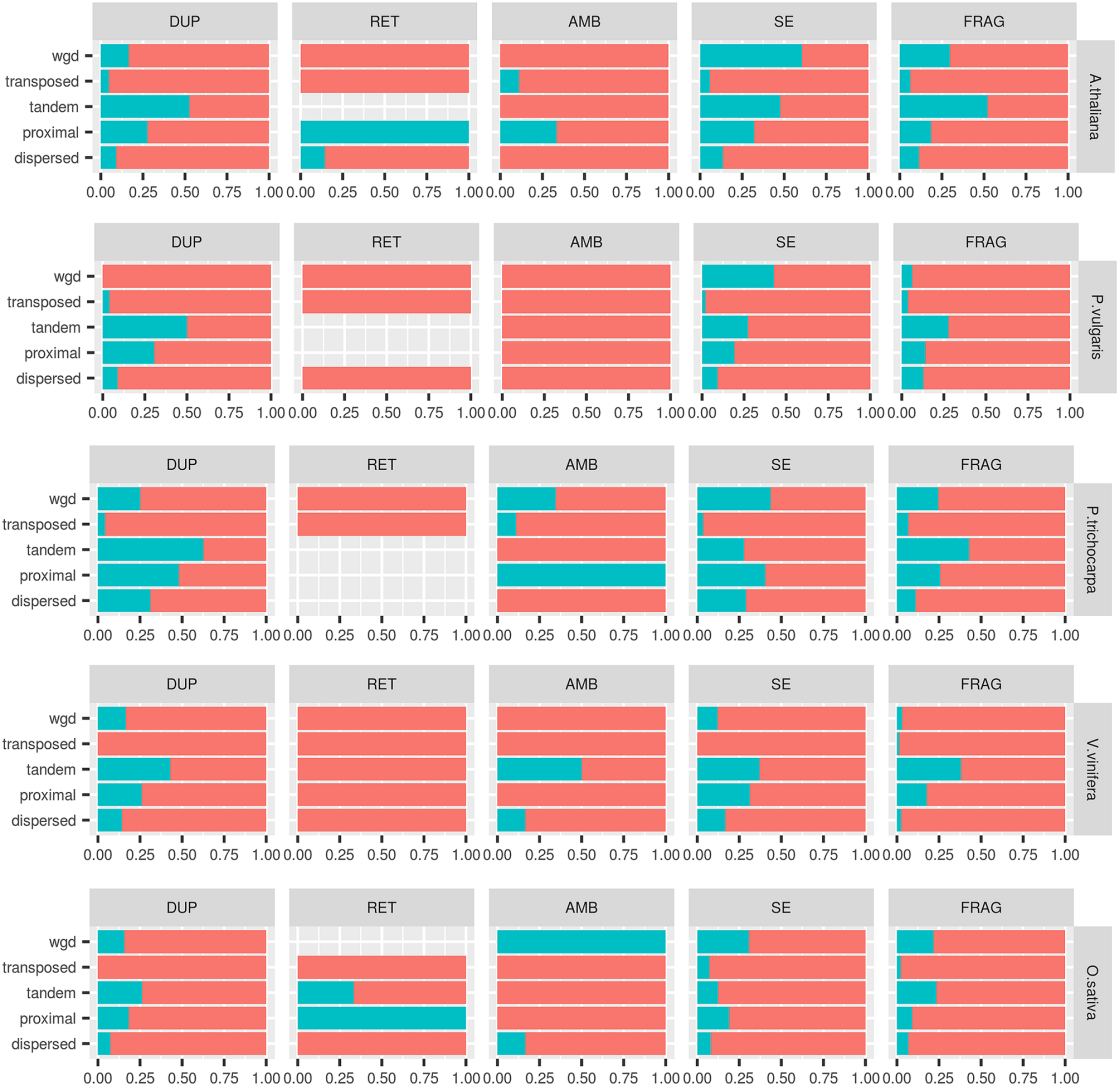
Pseudogene flanking regions with (blue) or without (red) homology to the correspondent parental loci sequences. Parental locus and pseudogene pairs were considered homologous at the flanking regions if at least one (either 5’ or 3’) of their correspondent flanking regions (2500 bp) aligned for more than 900 bp with an identity of at least 80%.

Next, we analyzed the relations between pseudogene structure and origin from parental loci. To avoid the uncertainties connected with the presence of multiple pseudogenes for a given parental locus we considered only cases of one to one relationships. In fact, in one (parental locus) to many (pseudogenes) relationships, it could be unwieldy to distinguish the exact origin of each pseudogene copy either deriving, directly, from duplication of parental locus (hereby referred as primary pseudogenes) or from an ancient pseudogene copy. Only duplicate pairs concordant with the pseudogene-parental locus (inferred) relationships were considered. Most pseudogenes were inferred as generated by transposition or dispersion (Fig. 5). An interesting exception was represented by *P. trichocarpa* pseudogenes which were prevalently originated by whole genome duplication. Pseudogenes classified as RET by intron-exon structure analysis were prevalently classified as transposed or dispersed. All duplication modes contributed to the formation of non processed pseudogenes, and their relative importance resembled the general duplication pattern. Of particular interest were the information for the type ambiguous, single-exon and fragmented since the classification based on gene structure is ambiguous or uninformative. The duplication modes of these types followed the general pattern with the exception of a higher frequency of transposition for the fragmented type. We did not observe a marked association between the presence of stretches of adenines and a specific pseudogene type or duplication mode (Figure S6). With the only exception of *A. thaliana* FRAG pseudogenes we did not see a clear preferential coverage for the 3’ end of parental locus transcripts (Figure S7). Since duplication in retroposed loci is restricted to exon sequences, no sequence homology to parental locus un-transcribed regions is expected^5,14^. Such a feature is optional for other duplication models. Indeed, we found that the evolution of pseudogene flanking regions varied with the duplication modes. Duplication by transposition was associated with the lowest occurrence of pseudogenes showing sequence homology in flanking regions (Fig. 6). Notably, these associations were confirmed for all pseudogene types and as expected were particularly strong for the RET type.* P. vulgaris* and *V. vinifera* showed the strongest effects compared to other species (Fig. 6). On the other side, pseudogenes originated by tandem duplication showed the highest tendency to preserve sequence homology in the flanking regions (Fig. 6). This observation was confirmed in all investigated species, for most pseudogene types and considering several thresholds of sequence homology (see Figure S8). Since several duplication mechanisms rely on the presence and/or activity of neighbour transposon elements^21^ we screened the flanking pseudogene sequences for homology to transposon elements. Transposed pseudogenes showed the highest associations to transposon elements in both *A. thaliana* and *V. vinifera* for all investigated thresholds of sequence (Figure S9).

### Pseudogene genomic distribution

**Figure 7:**
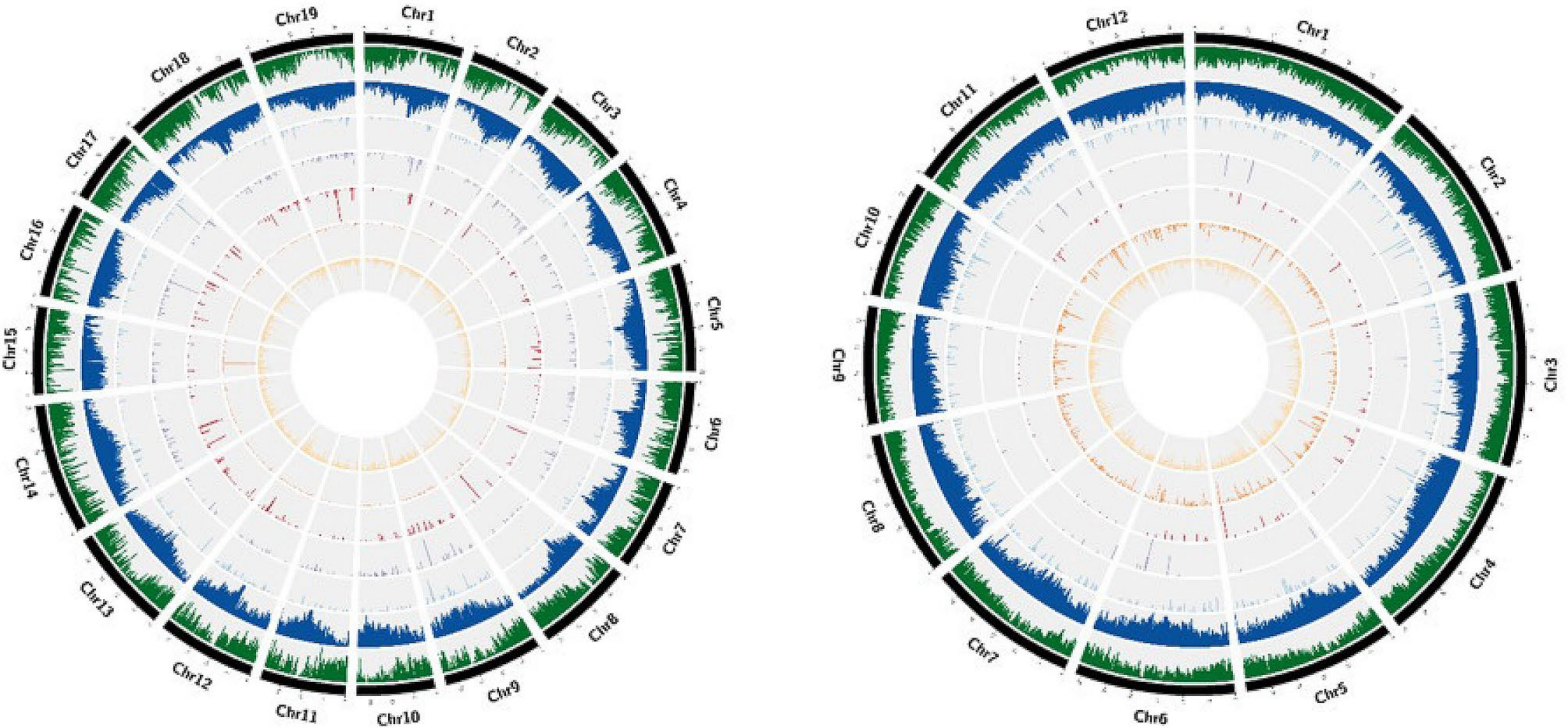
Circos graphs for distribution of genomic and features of the *V. vinifera* (left) and *O. sativa* (right) genomes. The histograms within each graph represent the densities of (from outer to inner): functional genes (green); transposon elements (blue); DUP pseudogenes; RET pseudogenes; AMB pseudogenes; single-exon pseudogenes; and FRAG pseudogenes.

We considered pseudogene density in adjacent chromosomes blocks of 1 Mbp and found that they were not distributed homogeneously along the five chromosome (*P*< 0.05 for goodness of fit test to a Poisson distribution). Next, we draw circos graphs to visualize pseudogenes distributions in relation to repetitive sequences and functional genes (Fig. 7). At the level of resolution of these graphs, no relevant association between densities of pseudogenes and repetitive sequences or genes were observed. All pseudogene distributions showed spikes of densities which represented stretches of sequence duplicated in tandem.

### Pseudogene structural and sequence evolution

**Figure 8:**
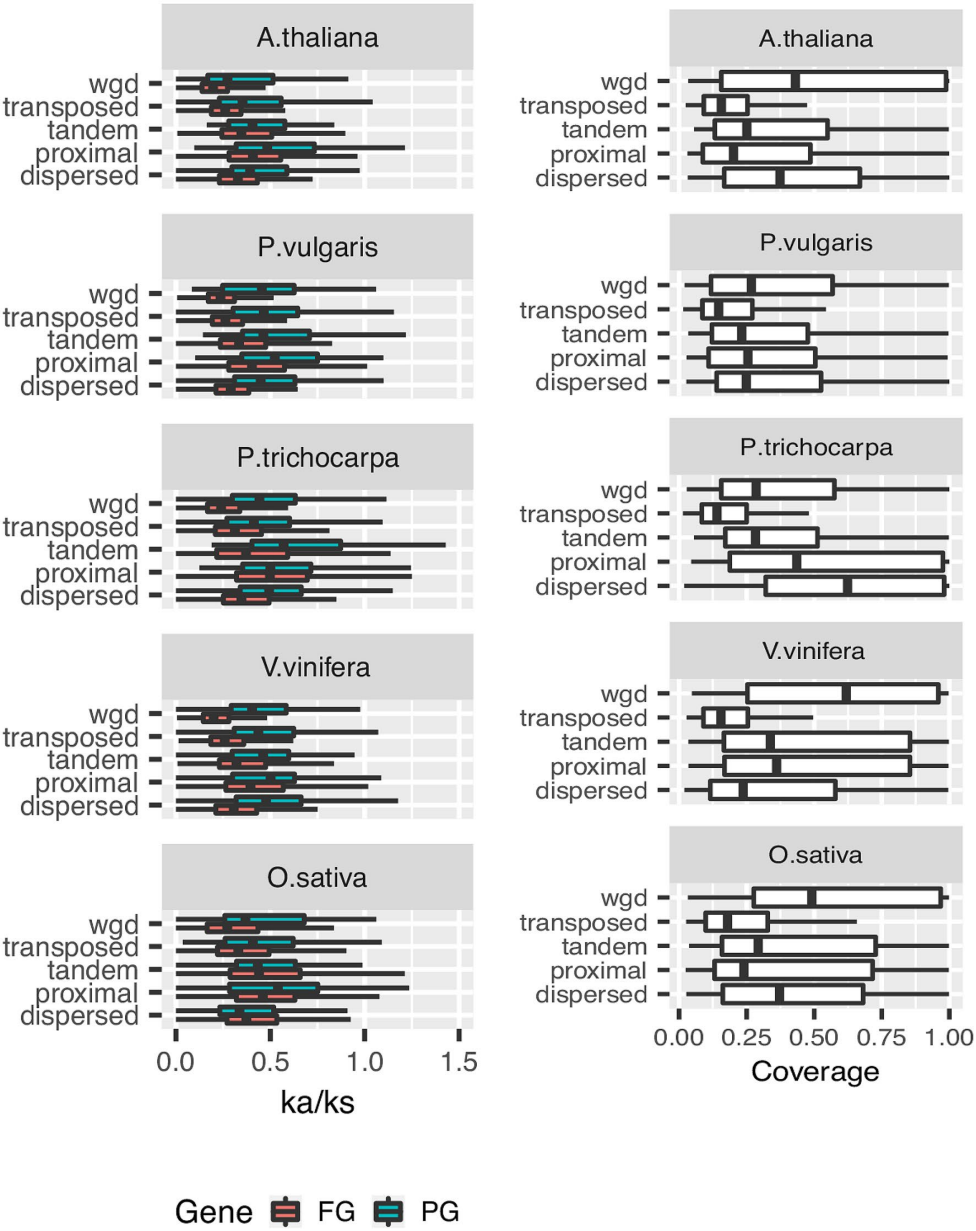
Evolutionary rates of the pseudogenes. (**a**) Comparisons of the omega ratios (ka/ks) calculated for the alignments of pairs of functional paralogs (blue) or pseudogene-parental locus pairs (red) in relation to the defined modes of duplication. (**b**) Coverage fractions of pseudogene protein alignments with the parental locus coding sequences.

Being inactive, pseudogenes are expected to evolve neutrally^1^. Accordingly, we found that the ratio between non-synonymous versus synonymous substitutions was significantly higher in alignments between pseudogenes and their functional parental loci (KW test, *P*< 0.001) than in those involving pairs of functional paralogous in all analyzed species. Evolutionary studies have suggested that the mechanism of gene duplication may influence the structural divergence between paralogous loci^22^. To verify if this observation holds also for pseudogenes-parental locus pairs the substitution rates were reconsidered after controlling for the duplication mechanism. The whole genome duplication mode (Fig. 8a) was associated with the highest purifying selection in both pseudogenes-parental loci and functional paralogous pairs. Little differences were noticed among the other duplication modes in both pseudogene-parental locus and functional paralogous pairs. As a measure of structural divergence, we considered the length of pseudogene aligning to the parental locus coding sequence. Pseudogenes generated by whole genome duplication showed the lowest structural divergence in most of the investigated species. In most species, transposed pseudogenes showed the shortest cds coverage (Fig. 8b).

### Pseudogene ancient functions

If inactivation by disabling mutation contributes significantly to copy number regulation of functional genes, we could expect an association between the family size of parental loci and the number of deriving pseudogenes. Indeed, positive associations between family sizes of the pseudogene and functional gene complements were observed in all investigated species (data not shown). The family definitions were based on protein functional domains (PFAM)^23^. Pseudogenes were considered to have the same domains of their parental loci even when parental locus to pseudogene alignments did not extend to domain regions. Several lines of evidence indicate that specific gene functions are subjected to a more finely tuned copy number regulation as illustrated by some families showing more (or less) than proportional size variations. To evaluate such hypothesis we compared relative abundances of pseudo and functional gene families. Table 1 reports the top functional domains which were over-represented in the investigated gene complements. Owing to the variability of the number of pseudogenes identified by each parental locus, it is possible that the observed domain enrichments have a different origin, for example from a widespread pseudogenization of most family members or an exaggerated pseudogene formation from only a few of them. To discriminate these options, we considered the family membership of parental loci instead of the number of generated pseudogenes (Table 1). This setting is more conservative for assessing family dynamics as some pseudogenes may derive from partial duplication of parental locus sequence or may be generated by pre-existing pseudogene copies. Several PFAM domains enriched in the pseudogene complements were also enriched in the parental gene complements (Table 1) suggesting that some gene families have a higher tendency to give rise to pseudogenes than others. Pseudogenes and functional proteins were clustered across the five investigated species based on aminoacid sequence identity. To reduce the noise due to short fragmented pseudogenes we considered for these analyses only sequences longer than 100 aa. Most of the identified clusters included pseudogenes from only one species (see supplemental figure S9 for details). On contrast, the clusters including at least one functional representative from each species were the most frequent (Figure S10). Ten clusters included pseudogenes from all investigated species. The most representative PFAM domains of these clusters included protein kinases (PF07714, PF00069), cell wall associated kinases (PF13947, PF08488), pentatricopeptide repeat containing proteins (PF01535), leucine rich repeat proteins (PF08263, PF00560), cytocrome P450 (PF00067) and photosynthetic reaction center containing protein (PF00124).

**Table 1:** Enrichment tests of pseudogene and pseudogene parental locus PFAM domains with respect to PFAM domains of functional loci used as queries across the five plant genomes.

Test	*A. thaliana*			*P. vulgaris*			*P. trichocarpa*			*V. vinifera*			*O. sativa*		
	PFAM	Family	Odds	PFAM	Family	Odds	PFAM	Family	Odds	PFAM	Family	Odds	PFAM	Family	Odds ratio
Pseudo versus functional	PF007723	Glycoside hydrolase	4.01	PF0076	RRM1	2.72	PF13676	TIR	2.53	PF00954	S-locus	7.34	PF14288	FKS1	22.48
	PF00646	Fbox	4.88	PF14111	DUF4283	10.48	PF13966	Zf-RVT	101.47	PF01453	B-lectin	3.61	PF02364	Glucan synthase	22.48
	PF08387	FBD	4.63	PF08626	TRAPPC9-Trs120	29.22	PF14111	DUF4283	8.28	PF00069	P kinase	1.4	PF04983	Rbp1	25.63
	PF07734	FBA	5.79	PF12739	TRAPP-1	29.22	PF05695	DUF825	48.45	PF00831	NB-ARC	21.45	PF04998	AKAP95	25.63
	PF08268	FBA-3	5.28	PF02889	Sec-63	7.5	PF05695	DUF825	48.45	PF00831	NB-ARC	21.45	PF04998	AKAP95	25.63
	PF08268	FBA-3	5.28	PF02889	Sec63	7.5	PF05970	PIF1	9.03	PF01582	TIR	77.87	PF13966	Zf-RVT	36.14
	PF14392	Zf-CCHC-4	13.21	PF02892	zf-BED	18.95	PF00560	LRR-1	1.78	PF05964	FYRN	12.4	PF13456	RVT-3	38.1
	PF14111	DUF4283	16.82	PF04937	DUF659	18.96	PF14392	Zf-CHHC-4	5.55	PF05965	FYRC	12.4	PF00069	*P*	Kinase 2.51
	PF13456	RVT-3	8.03	PF05964	FYRN	6.31	PF13855	LRR-8	1.55	PF00400	WD40	3.51	PF00623	RBp-1	20.5
Parental versus functional	PF07723	Glycoside hydrolase	5.49	PF00931	NB-ARC	5.21	PF00560	LRR-1	2.63	PF07714	P-kinase-Tyr	1.66	PF13855	LRR-8	3.10
	PF00646	F-box	7.37	PF14111	DUF4283	79.49	PF13855	LRR-8	2.35	PF00069	P-kinase	1.66	PF08263	LRRNT-2	3.12
	PF08387	FBD	6.46	PF13676	TIR-2	6.51	PF01535	PPR	2.34	PF08370	PDR-assoc	18.96	PF00069	P-kinase	3.11
	PF07734	FBA	9.87	PF08370	PDR-assoc	16.62	PF13041	PPR-2	2.26	PF00082	Peptidase-S8	7.73	PF00560	LRR-1	3.08
	PF08268	FBA-3	10.39	PF14510	ABC-trans	15.17	PF08263	LRRNT-2	2.31	PF01453	B-lectin	5.97	PF07714	Pkinase-Tyr	2.55
	PF12819	Zf-CCHC-4	5.95	PF13855	LRR-8	2.24	PF13676	TIR-2	4.22	PF00005	ABC-tran	2.96	PF12796	Ank-2	3.66
	PF01344	Kelch-1	4.51	PF01582	TIR	10.39	PF00931	NB-ARC	3.10	PF05922	Inhibitor-I9	7.02	PF08370	PDR-Assoc	18.52
	PF03478	DUF295	10.96	PF00560	LRR-1	2.17	PF14432	DYW	3.72	PF00122	E1-E2-ATPase	5.62	PF00005	ABC-tran	3.31

Fisher tests: alternative hypotheses for odds ratio>1 and *P* value<0.05, after Bonferroni correction for multiple testing (enrichment for specific PFAM domain in the pseudogene complement).

## Discussion

All systematic surveys so far carried out in plant genomes^7,8,10,12,24^ conclude that the majority of identified pseudogenes are fragmented. In addition, due to methodological differences among studies, no consensus view on the balance between pseudogene types in plant genomes has been reached. Zou and coworkers^24^ reported that in rice intergenes, pseudogenes retaining intron sequences are as abundant as those that, being generated by retroposition of spliced mRNA, are devoid of introns. In contrast, other reports have indicated that duplicated outnumbered processed pseudogenes in the grasses *O. sativa*^12^ and *H. vulgare*^10^. A recent survey in seven plant genomes^7^, including monocots and dicots, indicated that, on average, 25 % of identified pseudogenes retained introns but no information was provided about the origin of those lacking introns. We identified and classified pseudogene types by applying a two-step approach rooted on the exon-intron structure of parental loci. Pseudogenes were initially identified based on sequence homology to querying (translated) exons and, then, classified based on the preservation of the parental locus as genic exon-intron structure. Although stringent criteria were always used for parental locus assignment, we cannot rule out the possibility that some ambiguous parental locus pseudogene assignments still exist in our dataset, especially for gene families with several highly homologous paralogous. Another limitation we point out is that such approach partially approximate pseudogenes as if it were functional protein. Based on these analyses we confirmed that non processed are more abundant than processed pseudogenes in all investigated species, an observation in striking contrast to the high predominance of retroposed pseudogenes of mammalian genomes^4^. Nevertheless, the observed variations in both balance and genomic distributions of pseudogene types underlined evolutionary differences among investigated pseudogene complements. The ratio between non processed versus processed pseudogenes ranged from about ten in *O. sativa* to the minimum of about 2 in *V. vinifera*. The distribution of pseudogene types in coding and not coding genomic regions was nearly invariant among species with the exception of *V. vinifera* which showed an unusually high proportion of pseudogenes in non coding regions (introns and utr). Since retroposition is generally considered as the main source of processed pseudogenes and it is known to be mediated by both LTR and non LTR retrotransposon elements, abundance and composition of these mobile elements may account for the differences of pseudogenes types among species. The high prevalence of processed pseudogenes in humans is generally explained by the high activity and abundance of LINE elements^3^ which are thought to provide the enzymatic machinery for retro-transcription and integration^3,10,14^. Prade et al.^10^ argued that the low abundance of processed pseudogenes in barley could be explained with LINE elements reaching less than 1% of the genome though the other types of repetitive elements cover more than half of barley genomes. Most of the LINE elements identified in the plant genome belong to the L1 and RTE clade, with other clades being present only in traces^25^. The L1 class is generally the most abundant and shows a high sequence and domain organization variation while the RTE sequence is quite uniform^25^. It is therefore possible that the observed difference of processed pseudogene abundance could be associated with the diversity and abundance of L1 subfamilies in the analyzed species. For example, *V. vinifera* L1 repertoire presents a high abundance of the L1 PUR clade which is virtually absent in other plant genomes^25^. In addition, LINE elements show a clear preference for introns in *V. vinifera*^26^ and thus it is possible that a similar tendency holds for LINE derived pseudogenes. Genome wide searches conducted in rice have identified a high number of retrocopies^27^ leading to the hypothesis that retroposition is an important duplication route modelling rice genome. Similar observations were conducted in *P.trichocarpa*^15^ and *A. thaliana*^28^ although in these species retroposition resulted less frequent than in rice. The apparent contradictions between these data and our conclusions could be reconciled by taking into account the differences in the procedure for parental locus identification. We queried the genomes with all predicted functional loci while the other cited studies used only loci with multi-exon models so disregarding the possibility that retrocopies may derive from genomic duplication of intron-less genes. To evaluate this hypothesis we reconsidered parental locus identifications by resolving the cases of pseudogene loci matching both single exon and multiple exon parental loci in favour of the latter. The number of intron missing models classified as retroposed increased but not to the point of disproving our conclusions. Abdelsamad and Pecinka^29^ reported that, in contrast to animals where 82% of retrocopies contain premature stop codons, only 17.4% retrocopies are classified as retro-pseudogenes in *A. thaliana*. Similar balances between retrogenes and retro-pseudogenes were reported in rice^27^ and *P.trichocarpa*^15^ and these results are compatible with the low abundance of retro-pseudogenes observed in our and other genome wide pseudogenes searches^10^. To discriminate between duplication of intron-less parental loci or their retroposition we also analyzed two additional hallmarks or retroposition: the presence of polyA at the 3’ end and preferential sequence homology for the 3’ end of parental transcripts. Both these hallmarks were not discriminative in the investigated species with the exception of a moderate prevalence of a preferential sequence homology for the 3’ end of parental transcript in *A. thaliana* fragmented pseudogenes. Finally, we showed also that the classification of SE pseudogenes based on the analysis of collinear maps resembled that of duplicated pseudogenes. This observation led support to the hypothesis that most of the observed SE pseudogenes derive from genomic duplication of SE parental loci. Retroposed sequences are usually considered dead on arrival since they lack the regulatory sequence essential for gene expression. They could become functional upon recruitment of regulatory sequence from neighbour functional loci or by exaptation of non genic regions^27^. It is not clear whether the balance of retrogenes versus retro-pseudogenes observed in plants is the result of post-integration selection which leads to rapid loss of non-functional sequence or to a higher density of cryptic promoters in plant genomes which allows a more efficient regulatory sequence recruitment. We integrated the classification of pseudogenes based on structure in terms of intron-exon organization and flanking sequence conservation with the inferred duplication mode. This approach highlighted associations between pseudogene structure and duplication mechanisms. For example, we found that the proportion of pseudogenes showing homology to parental flanking regions followed a trend with tandem>wgd>proximal>>transposed/dispersed. Noteworthy these observations were reproduced for most pseudogene types and thus they cannot be explained with retro-pseudogenes being prevalently classified as transposed and expected to show no homology to parental flanking regions. We noticed also that on average the number of pseudogenes classified as transposed showed a parental loci cds coverage shorter than those classified otherwise. Both these observation are compatible with a model of sequence duplication based on Double Strand Break Repair (DSBR) mediated by Non homologous End Joining (NHEJ) via Synthesis Dependent Strand Annealing (SDSA) in which gene fragments are copied as filler DNA at break sites induced by transposon or by other accidental causes. Buchanan et al.^30^ have demonstrated that sequences flanking gene fragments duplicated via NHEJ-SDSA evolve rapidly. Instead, duplicated copies inferred as proximal or tandem are subjected to concerted evolution to a higher degree than those that are located near one another. Qiao et al.^20^ have demonstrated that classes of gene duplicates show distinct patterns of temporal and functional evolution^20^. WGD duplications are more conservative with smaller ka/ks rates than tandemly and proximal duplicates suggesting that they have experienced long-term purifying selection. On the contrary, tandem and proximal duplicates resulted more subjected to positive selection and functional diversification. In addition, analysis of gene-ontology of duplicated genes suggested that whole genome duplications contributed more to balanced changes of entire pathways or developmental processes while tandem and proximal duplications was somehow more dedicated to generate novel variant with adaptive potential. Based on these findings the authors proposed that whole genome duplication produces duplicates with longer half-lives than tandem and proximal duplicates. Our comparative analyses of pseudogene and functional gene complements confirmed such a scenario. In all investigated species we found proportionally less whole genome and more proximal and tandem duplicates among pseudogenes than functional loci. Such a finding could be explained also with the hypothesis of tandem and proximal duplication modes being more prone to generate disabled copies than whole genome duplication. If so we should expect tandem and proximal pseudogenes clearly associated to fragmented types and tendentially showing no sequence homology to parental locus flanking regions. The analysis of a dataset of primary pseudogenes did not provide either of these evidence. Instead we found both these evidence in duplicates classified as dispersed. Notably, in all analyzed species the dispersed duplicates were proportionally more abundant in the pseudogene than in the functional gene complement. The PFAM domains overrepresented in parental loci are common in proteins involved in plant defense (for example LRR, NB-ARK, TIR S locus glycoprotein) cell wall associated transporters (ABC transporters), and protein degradation mechanisms (F-box). Most of these genes families contribute to the high dynamic responses of plants to both biotic and biotic stresses^24^. The presence of an excess of pseudogenes could indicate their high duplication and turnover rates. Specific studies with dedicated pipelines will clarify whether these pseudogenes interact with their functional cognate by regulating their expression or sequence evolution^1,15^.

## Methods

**Sequence and annotation datasets** Sequence and annotation files were downloaded from the Phytozome web site (https://phytozome.jgi.doe.gov/pz/portal.html), except for *V. vinifera* which was downloaded from the CRIBI web site (https://genomes.cribi.unipd.it). The genomic coordinates of functional loci were obtained as general feature format (gff) files downloaded from phytozhome. PFAM information^23^ were obtained as part of the genomic annotation_info.txt files which were downloaded from phytozome. Genomic sequences were masked using Repeatmasker version open-4.0.5^31^ using species specific libraries of repeat elements. For all investigated species, genome sequences with “chromosome” assembly levels and gene predictions, validated through RNAseq assemblies and ESTs, were used. A list of ftp addresses and downloaded files is reported in supplemental Table S1.

**Pseudogene identification** Pseudogenes were identified by applying the pipeline outlined in Fig. 1^32^. In brief, peptide sequences corresponding to coding exons were used as queries for tBlastN^19^ searches against the hard masked version of genomic sequence. Coding sequences (CDS) matching for at least 30% of length to a repetitive sequence were not considered for downstream analyses. Coding exon sequences were extended at both sides to include additional nucleotides from neighbouring regions, a modification hereby referred to as “exon tail”. Depending if the exon started with the first, second or third nucleotide of an in frame codon, the tail length was 51 52 or 53 bp, respectively. All the hits with an identity higher than 40% and an e-value lower than a 1e-6 were considered for further analysis. Hits matching the same query sequence and with overlapping coordinates were merged to give rise to super-hits. Neighbouring super-hits that matched the same query sequence were merged if their distance on the chromosome differed by the corresponding distance on the query sequence by less than 100 bp^6^. These hits were considered to belong to the same pseudo-exon and the intervening gap generated by low complexity or very decayed regions, short insertions within the pseudogene or repetitive elements^6^. Super-hits that matched adjacent exons of the same locus were merged when their distance on the chromosome differed by not more than 5000 bp from the length of the corresponding intron (i.e the intron between the two matching exons). Super-hits that mutually overlapped for more than 20% of their respective lengths were clustered in the so called pseudogenic regions. For each region, the best pseudogene-query (functional) locus pairs was selected based on the e-value and tBlastN score (before hits merging)^6^. The selected functional locus was considered as pseudogene “parental locus”, i.e the locus which generated the pseudogene, by assuming its actual sequence as the best available approximation of the locus sequence at the time of pseudogene formation^6^. Genomic coordinates of pseudogene and functional gene models were compared with bed tools^33^ software to identify the pseudogenes which map within introns, UTR or intergenes.

**Prediction of pseudogene models and pseudogene classification** The full length protein sequences of the parental loci were aligned to the target pseudogenic sequences using Genewise^34^. This software aligns protein to DNA sequences and infers intron-exon splice sites while allowing stop codons and frameshift. Nucleotide sequence homology between parental locus introns and pseudogenic regions was computed by BlastN^19^ as reported in the Hopssigen protocol^35^. The genic model of functional genes were obtained from gff files using gff2sequence^36^. The pseudogene and parental locus models were compared to check the position of inferred intron-exon splice sites and classify pseudogenes. Non processed pseudogenes originate from chromosomal or segmental duplication and thus retain the intron-exon structure of parental loci. Under these circumstances the positions of introns in the pseudogene and the parental locus should match. These matching positions were coded as ‘dup’(Figure S1a). Intron positions showing uninterrupted stretch of gaps in the parental locus, though no introns were predicted in the pseudogene by Genewise^7^, were also coded as ‘dup’. The opposite situation: i.e. gaps in the pseudogene at positions where introns are expected, were coded as ‘amb’ to underline that no definitive conclusion could be taken on the pseudo-intron (Figure S1b). Finally, intron positions in the parental locus showing no hallmark of predicted intron in the pseudogene were coded as ‘ret’ (Figure S1c). The pseudogenic regions identified by super-hits matching adjacent exons and for which Genewise could not predict a reliable model were classified based on hits relative distance according to Xie et al.^7^. A ‘dup’ code was inferred when pair of hits identified by adjacent parental locus exons were separated by more than 50 bp. If the distance between hits was lower than 50 bp and the pseudogenic regions did not show homology to parental locus introns the position was coded as ‘ret’ unless homology to parental locus intron sequence could be demonstrated in which case the code was changed to ‘amb’. Pseudogenes models with all intron positions coded as ‘dup’ were considered as duplicated. Those showing all intron position classified as ‘ret’ and none as ‘dup’ or ‘amb’ were classified as processed if no nucleotide homology to parental locus intron sequence was detected. The other cases: i.e. pseudogenes with both ‘dup’ and ‘ret’ intron positions or with ‘amb’ were classified as ambiguous. Pseudogenes that showed homology to parental loci with single coding exon models were classified as single exon pseudogene. We classified as fragmented all the cases in which the alignments between pseudogene and parental locus did not cover any intron positions. The poly A tail of pseudogenes, a hallmark of retroposition, were identified as genomic regions showing more than a minimum number of consecutive adenines while tolerating only one mismatch^29^. The minimum number of adenines was identified by considering the frequency distribution of stretches of consecutive adenines (with a single mismatch) within 600 bp at the 3’ flanking region of non processed pseudogenes. For each species, we defined this adenine minimum number as the value which left out 5% of the distribution^29^. The minimum number was set to 15 for Arabidopsis and 14 for the other species. Finally, we used tfasty36 to identify disablements (frameshift or stop codons) in sequence alignments of the parental protein to pseudo-exon sequence.

**Evolutionary rate calculation** The level of evolutionary constraints of pseudogenes was inferred by calculating the synonymous and non-synonymous substitution rates between each pseudogene and the parental locus sequence. The ratios were determined using the yn00 software in the PAML package^37^ on coding sequences which were reconstructed based on pseudogene-parental protein alignments. Sites corresponding to stop codons, frameshifts or gaps were ignored.

**Analysis of gene ontology and family enrichment** Pseudogenes truncated and or degenerated were predicted to have the same domains of the parental loci. We performed a classical enrichment analysis by testing the over-representation or under-representation of PFAM terms using Fisher’s exact test using fisher.test function as implemented in R (Core Team, 2013).

**Synteny among chromosomes and duplicate classification.** Sequence duplicates were classified using DupGenefinder^20^. Briefly, all query proteins of each species were compared with proteins of the other investigated species and with themselves using BlastP^19^ and the best five non-self matches with an e-value below 1e-5 were reported. The resulting hits were studied according to the position of the genes in the chromosomes and scaffolds. The highest scoring path was identified by dynamic programming as implemented in DupGeneFinder with standard settings and inter and intra species maps including both functional and pseudogene loci were constructed. *Oryza sativa* was used as outgroup for analysis using the other species as targets whereas *Spirodela polyrhiza* was the outgroup for the analyses involving *O. sativa* as target species. To avoid possible conflicts due to overlapping gene coordinates we considered only intergenic pseudogenes. Pairs of homologous sequences were classified as segmental if they were collinear. Pair of homologous sequences were classified as tandem or proximal, if they mapped at adjacent or close (separated by less than 25 intervening loci) respectively. The remaining loci, after removing the above categories, were considered as transposed if at least one partner of the pair was a collinear gene in a interspecies collinear map or otherwise as dispersed. The cases of pseudogenes being collinear genes and the parental loci transposed were not considered as these represent cases of unitary pseudogenes which will be characterized elsewhere.

**Analysis of pseudogene and parental loci flanking regions** The pseudogene 5’ and 3’ genomic flanking regions were extracted from unmasked genomic fasta file for the analysis of several pseudogenes hallmarks. The length of pseudogene flanking regions were calculated considering the length of either the 5’ UTR or 3’ UTR parental locus regions and the adjacent genomic regions whose length depended on the specific analysis. The length of flanking regions was calculated by adding the length of either 5’ UTR or 3’ UTR to an additional genomic regions. For the analysis of sequence homology between parental loci and pseudogene flanking regions the length of the adjacent genomic regions was set to 2500 bp. Correspondent pseudogene and parental loci flanking regions were aligned with lastZ^38^. A pseudogene-parental locus pair was considered as showing homology in flanking regions if at least one lastZ alignment was longer then a given threshold and presented at least 80% of identical positions. Several thresholds of alignment length were considered and a summary of results are reported in Supplementary Figure 8. For the analysis of transposon content in the flanking regions of pseudogenes we considered adjacent genomic regions of 500 bp. A pseudogene flanking region was considered as showing homology to a transposon element if it aligned to the transposon sequence for more than a given portion of the total query length and with an alignment e-value below e-10.

**Circos graphs** A global representation of the genomic landscape was produced for each species by using Circos version 0.69-9^39^. The chromosomes were divided into 200 kbp-window regions. For each region, the number of genes and TEs bases were calculated, as well as the number of bases covered by pseudogenes. Pseudogene genome distributions were analyzed according to their classification.

**Pseudogenes and functional loci clustering** Pseudogene encoded peptides and functional loci from the investigated species were clustered based on sequence homology using cd-hit^40^. The threshold identity level was set to 0.4 and sequences shorter than 100 aa were not considered.

## Supplementary information

Supplementary Table S1.

Supplementary Table S2.

Supplementary Table S3.
