# Supplementary material for: Structural characterization and duplication modes of pseudogenes in plants
Source: Sci Rep. 2021 Mar 5;11:5292. doi: 10.1038/s41598-021-84778-6 (PMC7935947; doi:10.1038/s41598-021-84778-6)
Supplement: Supplementary file 3 — Supplementary Table S3. [file 41598_2021_84778_MOESM3_ESM.docx]

Supplemental Table S3 of

“Structural characterization and duplication modes of pseudogenes in plants

Flavia Mascagni1, Gabriele Usai1, Andrea Cavallini1 and Andrea Porceddu2*

1Department of Agricultural, Food, and Environmental Sciences, University of Pisa, Via del Borghetto 80, I-56124

Pisa, Italy.

2Dipartimento di Agraria, Universit `a degli studi di Sassari via Enrico de Nicola 1. 07100 Sassari. Italy

*aporcedduuniss.it

a)

| Species | DUP | RET | AMB | SE | FRAG | Tot |  | DUP/RET |
| --- | --- | --- | --- | --- | --- | --- | --- | --- |
| A.thaliana | 556 | 60 | 118 | 1103 | 2838 | 4675 |  | 9.27 |
| P.vulgaris | 5354 | 275 | 1675 | 5778 | 21332 | 34414 |  | 19.47 |
| P.trichocarpa | 2999 | 227 | 682 | 6109 | 16190 | 26207 |  | 13.21 |
| V.vinifera | 2113 | 1127 | 705 | 4371 | 14462 | 22778 |  | 1.87 |
| O.sativa | 1298 | 78 | 230 | 1527 | 5332 | 8465 |  | 16.64 |

b)

| Species | DUP | RET | AMB | SE | FRAG | Tot |  | DUP/RET |
| --- | --- | --- | --- | --- | --- | --- | --- | --- |
| A.thaliana | 603 | 81 | 127 | 781 | 3394 | 4986 |  | 7.4 |
| P.vulgaris | 4857 | 374 | 1476 | 3535 | 25777 | 36019 |  | 13.0 |
| P.trichocarpa | 3050 | 278 | 711 | 4410 | 18951 | 27400 |  | 11.0 |
| V.vinifera | 2032 | 1257 | 722 | 2890 | 16524 | 23425 |  | 1.6 |
| O.sativa | 1307 | 100 | 229 | 1157 | 5940 | 8733 |  | 13.1 |

**Supplemental Table S3**

Pseudogene classification based on intron-exon structure

1. Classification based on reconstructed pseudogene models considering the information of the functional loci showing the highest structural and sequence homology.
2. Classification based on reconstructed pseudogene models. Cases of pseudogenes matching both a multiexon and single exon paters were ‘manually’ resolved in favour of the formers.
